# The College of Nursing, Limited

**Published:** 1917-05-05

**Authors:** 


### THE COLLEGE OF NURSING, LIMITED.

### Constitution of the Irish Board. Approved by the College.

1. That a Local Board for Ireland, to be designated the Irish Board, be established for the superintendence of the affairs of the College of Nursing in Ireland.

2. That the first Irish Board be composed of thirty members. [Here follow the names of twenty-two members which we have already published (see The Hospital, March 17 and April 28).] The remaining eight members to be co-opted.

3. That the Irish Board nominate six members to represent Ireland on the Council of the College.

4. Should any casual vacancy occur on the Board, the Board shall elect a person to hold office for the remainder of the period during which the member vacating his appointment would have held office.

5. That every Standing Committee of the Council of the College shall include at least one Irish representative in six.

6. That the Irish Board shall be free to regulate its meetings and proceedings subject to the approval of the Council.

7. That the Irish Board shall from time to time report to the Council through the Consultative Committee what hospitals in Ireland possess Nursing Schools whose training may be accepted as qualifying for the Register of the College.

8. That the Irish Board shall report to the Council, through the College Registration Committee, as to the qualifications of applicants for membership of the College, who have already completed their training in Ireland, or may complete their training during the period of grace.

9. That all examinations in Irish Nursing Schools shall be conducted in conformity with the standard of examination determined by the College Council by examiners appointed by the Council upon the nomination of the Irish Board through the Examination Committee.

10. That the Irish Board shall be provided with a local secretary resident in Ireland, and with an office, the expenses to be defrayed by the College. The secretary shall be appointed by the Council upon the nomination of the Irish Board, and shall carry out such duties as the Board may arrange.

11. That the Irish Board shall annually prepare and send to the secretary of the Finance Committee an estimate under various heads of the moneys required for expenditure by the Board, including travelling expenses of members to London.

12. That the estimate, when approved by the Council, and the amounts included therein, shall be appropriated to the several objects specified in the estimate, and the Irish Board shall be authorised to expend sums not exceeding such amounts during the financial year for the several objects so specified. No expenditure shall be incurred by the Board except such as is provided for in the annual vote. Any proposed expenditure not so provided for shall be the subject of a special estimate submitted by the Irish Board to the Council through the Finance Committee.

13. That periodical returns of the expenditure incurred shall be submitted to the Finance Committee, and the Finance Committee shall present to the Council at the termination of each financial year a statement thereon, with such comments as they may think advisable.

